# Risk factors associated with non-vaccination in Gambian children: a population-based cohort study

**DOI:** 10.1093/trstmh/trac051

**Published:** 2022-06-13

**Authors:** Benjamin Young, Golam Sarwar, Ilias Hossain, Grant Mackenzie

**Affiliations:** MRC Unit The Gambia at LSHTM, Fajara, 00000, The Gambia; London School of Hygiene and Tropical Medicine, London, WC1E 7HT, United Kingdom; MRC Unit The Gambia at LSHTM, Fajara, 00000, The Gambia; London School of Hygiene and Tropical Medicine, London, WC1E 7HT, United Kingdom; MRC Unit The Gambia at LSHTM, Fajara, 00000, The Gambia; London School of Hygiene and Tropical Medicine, London, WC1E 7HT, United Kingdom; MRC Unit The Gambia at LSHTM, Fajara, 00000, The Gambia; London School of Hygiene and Tropical Medicine, London, WC1E 7HT, United Kingdom; Murdoch Children's Research Institute, Melbourne, VIC 3052, Australia; Department of Paediatrics, University of Melbourne, Melbourne, VIC 3010, Australia

**Keywords:** immunisation programmes, immunisation schedule, The Gambia, vaccination coverage, vaccines

## Abstract

**Background:**

We determined the risk factors associated with unvaccinated children in rural Gambia.

**Methods:**

We conducted prospective demographic surveillance and recorded immunisations in real time in the Upper River Region, The Gambia. Analysis included residents born from 1 January 2012 to 31 December 2016. Data included age, sex, household members and relationships, migrations, births, deaths, ethnicity, residential location and birth type. Children were defined as unvaccinated at 10, 15 and 24 mo of age if they missed all primary series doses (pentavalent, oral polio and pneumococcal conjugate vaccines), secondary series (first dose measles and yellow fever vaccines) or both vaccination series, respectively. Logistic regressions measured the association between risk factors and being unvaccinated.

**Results:**

In total, 5% (1567/30 832) of infants born during the study period and who were residents at the age of 10 mo were unvaccinated. Being unvaccinated at 10 mo of age was associated with children; who did not reside with either parent (adjusted OR 2.26, 95% CI 1.60 to 3.19), whose parents were not the head of household (1.29, 1.09 to 1.52), who had experienced immigration (2.78, 1.52 to 5.08) or who were not of Mandinka ethnicity (between 1.57 and 1.85 for other ethnicities).

**Conclusions:**

Family characteristics are associated with unimmunised children in rural Gambia. Our findings may inform strategies to increase vaccine coverage.

## Introduction

Vaccines have been considered the most successful and cost-effective tool against infectious diseases.^1^ To ensure that vaccines are freely available to every child, the Expanded Programme on Immunisation (EPI) was established nearly 50 y ago by the WHO.^2^ Vaccination was estimated to save >20 million lives from 2001 to 2020.^3,4^ Despite this, delayed or missed immunisations in low- and middle-income countries (LMICs) lead to >2 million annual vaccine-preventable child deaths.^5–7^

The Global Vaccine Action Plan 2011–2020 aimed to increase national vaccination coverage worldwide to >90% for all EPI vaccines by 2020.^8^ In 2019, global vaccination coverage of diphtheria-tetanus-pertussis (DTP) vaccine's third dose reached 85%.^9^ However, high global coverage obscures the fluctuation and inequitable stagnation of progress in LMICs, as over the past decade nearly 20 million children remained unprotected annually, almost half residing in Africa.^9,10^ In LMICs and Africa especially, vaccination access varies substantially; in West Africa alone coverage varies from 57% in Nigeria to 88% in The Gambia.^9,10^

From 2009, the Gambian EPI included BCG vaccine, hepatitis B vaccine (HepB), oral polio vaccine (OPV), pneumococcal conjugate vaccine (PCV), DTP, conjugate *Haemophilus influenzae* type B vaccine (Hib), measles vaccine and yellow fever vaccine.^11^ In 2015, the Gambian EPI added rotavirus vaccine and rubella vaccine, a combination vaccine with measles vaccine, while group A meningococcal conjugate vaccine was added in 2019 (Supplementary Table A1).^11,12^ Vaccines are administered at Reproductive and Child Health (RCH) clinics.

The Gambian EPI has been a success story and model immunisation programme in sub-Saharan Africa (SSA), with generally high coverage since 1990.^13–15^ Part of its success has been due to high public awareness, with accessibility through permanent outreach sites for remote areas and static RCH clinics.^11,14^ To obtain high vaccination coverage, not only is a reliable immunisation programme necessary, but also usage of the programme by caregivers.^16^ The Gambia, with a well-established vaccination programme, represents a unique setting for research into the characteristics of families who do not utilise a successful programme.

A delay in age-appropriate vaccination in The Gambia occurs in two-thirds of children.^13^ Delayed immunisation is associated with the employment status of the mother, birthplace, transportation method and parental literacy.^2,13^ While researchers often study delayed vaccination,^17^ to the best of our knowledge, we are unaware of African studies reporting risk factors for being fully unvaccinated.

Studies have found that children of internal migrants or parents with low education are less likely to be vaccinated.^18^ Distance to the immunisation facility,^19^ parental age, the presence of the parents and their marital status, have also been associated with ‘under-vaccination’.^20,21^ Reluctance of parents to vaccinate their children is on the rise in high-income countries.^20^ However, vaccine hesitancy is less prevalent in LMICs.^20^

We aimed to identify factors associated with children being fully unvaccinated for the primary series antigens (OPV, PCV and the Pentavalent combination vaccine [DTP-HepB-Hib]) at 10 mo of age, the secondary series antigens (first dose measles and yellow fever) at 15 mo of age and for both series of antigens at 24 mo of age. We hypothesised that children living further from RCH clinics, immigrants and those with less educated or absent parents were more likely to be unvaccinated.

## Methods

### Study population

This was a population-based cohort study including residents of the Basse Health and Demographic Surveillance System (BHDSS). The BHDSS is located south of The Gambia River in the Upper River Region in eastern Gambia (population 177 853 in 2014), spread across 219 settlements. BHDSS residents born from 1 January 2012 to 31 December 2016 were included in the study and followed for 24 mo; those resident in the BHDSS at 10, 15 and 24 mo of age were included in the respective analyses to ensure appropriate time to be vaccinated.

### Data collection

Demographic surveillance in the BHDSS was conducted through household visits at 4-mo intervals. Data on births, deaths, migrations, household location, education level, head of households and composition of families were collected at each visit. Immunisation data were electronically recorded in real time at RCH clinics. A household socioeconomic status (SES) survey was conducted in 2012 based on the number of assets and livestock.

### Statistical analysis

We defined the primary series as three doses each of OPV, PCV and Pentavalent vaccine. At 10 mo of age, children were categorised as non-vaccinated if they had not received any doses of the primary series vaccines and vaccinated if they had received one or more doses of any primary series vaccine. The secondary series vaccinations were defined irrespective of the primary series antigens and assessed at 15 mo of age, including the first dose of measles vaccine and yellow fever vaccine. At 24 mo of age, we categorised children as unvaccinated if they had not received any of their primary or secondary series vaccinations. The birth series vaccination was ignored because we were concerned with the characteristics associated with non-vaccination in infancy. Given our period of follow-up, we ignored vaccines that were introduced after 2012.

The distance between households and RCH clinics was calculated using ArcGIS (version 10.5, Redlands, CA, USA) and categorised as distance <0.5, ≥0.5–<1, ≥1–<2, ≥2–<3, ≥3–<4 and ≥4 km. Residents were grouped by type of immigration: none, migration within the BHDSS, internal migration (within The Gambia) or external migration (from outside The Gambia), for each age point of interest. Date and location of residence and associated household headship were used to determine parental presence for the child and the child's relationship to the head.

Families were determined based on shared mother IDs and used to determine the birth order of children and type of pregnancy (twin or singleton). The highest educational level achieved by each parent was expressed as: none, basic, secondary, college, Koranic, Madrassa or other. The child's ethnicity was grouped based on local prevalence: Fula, Serahule, Mandinka and other. Finally, the mother's age at birth was divided into age groups, age <15, ≥15–<20, ≥20–<30, ≥30–<40 and ≥40 y.

Three-level mixed effects logistic regressions,^22^ adjusted for nested clustering within families and households, were used throughout the primary and secondary, univariate and multivariate analysis to produce crude and adjusted ORs (cOR and aOR) with 95% CIs and two-sided p-values using Wald's test. Univariate analyses assessed the distribution of risk factors, prevalence of endpoints and cOR (including a priori variables sex and mother's age at birth) to measure the crude association. All covariates that were weakly associated (p<0.20) with the exposure (Table 1), the endpoints and not on the causal pathway were defined as potential confounders in multivariate analyses. The aOR (including a priori variables and potential confounders) evaluated the adjusted association between risk factors and vaccination status (Table 1 and Supplementary Table A2). If multicollinearity between strongly associated controlling variables was evident (log of standard errors changes >10), the variable with the least effect on the effect estimate was removed.

**Table 1.** Primary analysis of characteristics of children within the BHDSS and crude and adjusted odds of being unvaccinated^I^ with the primary series of vaccination at 10 mo of age. Selected risk factors of interest are in bold

Descriptive variable^II^	TotalN= 30 832	(Col %)	Unvaccinatedn=1567	(Row %)	Crude OR^III^ (95% CI)	p-value^IV^	Adjusted OR (95% CI)	p-value^IV^
Sex								
Female	15 076	(48.9)	775	(5.1)	1		ND	ND
Male	15 753	(51.1)	792	(5.0)	1.00 (0.89 to 1.12)	0.94		
Missing	3	(0.0)	0	(0.0)				
**Ethnicity** ^V^								
Mandinka	6418	(20.8)	231	(3.6)	1	<0.001	1	<0.001
Fula	9836	(31.9)	525	(5.3)	1.57 (1.31 to 1.89)		1.61 (1.33 to 1.96)	
Serahule	14 070	(45.6)	782	(5.6)	1.64 (1.38 to 1.95)		1.58 (1.32 to 1.90)	
Other	505	(1.6)	29	(5.7)	1.85 (1.19 to 2.89)		1.85 (1.14 to 3.00)	
Missing	3	(0.0)	0	(0.0)				
**Distance to RCH** ^VI^								
≥0 and <0.5 km	15 113	(49.0)	737	(4.9)	1	0.26	1	<0.001
≥0.5 and <1 km	4616	(15.0)	263	(5.7)	1.21 (1.02 to 1.44)		1.23 (1.03 to 1.46)	
≥1 and < 2 km	4502	(14.6)	240	(5.3)	1.10 (0.92 to 1.31)		1.16 (0.96 to 1.40)	
≥2 and <3 km	3051	(9.9)	151	(4.9)	1.01 (0.82 to 1.26)		1.05 (0.84 to 1.31)	
≥3 and <4 km	2136	(6.9)	95	(4.4)	0.92 (0.71 to 1.18)		0.96 (0.74 to 1.25)	
≥4 km	283	(0.9)	16	(5.7)	1.11 (0.62 to 1.98)		1.10 (0.68 to 2.06)	
Missing	1131	(3.7)	65	(5.7)				
**Migration** ^VII^								
No in-migration	28 404	(92.1)	1402	(4.9)	1	<0.001	1	<0.001
Within the BHDSS	551	(1.8)	43	(7.8)	1.80 (1.26 to 2.58)		1.67 (1.15 to 2.44)	
Internal in-migration	503	(1.6)	33	(6.6)	1.39 (0.91 to 2.13)		1.22 (0.79 to 1.89)	
External in-migration	177	(0.6)	21	(11.9)	2.90 (1.61 to 5.24)		2.60 (1.39 to 4.87)	
Missing	1197	(3.9)	68	(5.7)				
Birth order								
First	22 223	(72.1)	1108	(5.0)	1	0.67	ND	ND
Second	7485	(24.3)	362	(4.8)	0.98 (0.86 to 1.12)			
Third	601	(1.9)	36	(6.0)	1.25 (0.85 to 1.83)			
Fourth or higher	30	(0.1)	2	(6.7)	1.24 (0.23 to 6.81)			
Missing	493	(1.6)	59	(12.0)				
Pregnancy type								
Singleton	29 324	(95.1)	1452	(5.0)	1		ND	ND
Twins	1015	(3.3)	56	(5.5)	1.09 (0.75 to 1.59)	0.65		
Missing	493	(1.6)	59	(12.0)				
**Head of house** ^VIII^								
Was a parent	6020	(19.5)	247	(4.1)	1		1	
Was not a parent	24 471	(79.4)	1298	(5.3)	1.31 (1.12 to 1.53)	<0.001	1.29 (1.09 to 1.52)	<0.001
Missing	341	(1.1)	22	(6.5)				
Mothers age at birth, y								
<15	178	(0.6)	10	(5.6)	1	0.26	ND	ND
≥15 and <20	3763	(12.2)	194	(5.2)	0.93 (0.46 to 1.89)			
≥20 and <30	16 244	(52.7)	838	(5.2)	0.93 (0.47 to 1.86)			
≥30 and <40	8799	(28.5)	399	(4.5)	0.80 (0.40 to 1.61)			
≥40	1338	(4.3)	67	(5.0)	0.89 (0.42 to 1.87)			
Missing	510	(1.7)	59	(11.6)				
**Presence of parents** ^IX^								
Both present	13 938	(45.2)	574	(4.1)	1	<0.001	1	<0.001
Mother present	15 605	(50.6)	867	(5.6)	1.40 (1.25 to 1.58)		1.38 (1.22 to 1.58)	
Father present	155	(0.5)	12	(7.7)	2.78 (1.32 to 5.88)		2.93 (1.33 to 6.46)	
Neither present	1134	(3.7)	114	(10.1)	2.26 (1.62 to 3.15)		2.26 (1.60 to 3.19)	

Abbreviations: BHDSS, Basse Health and Demographic Surveillance system; RCH, Reproductive and Child Health centre.

^I^Children were defined as unvaccinated if they had not received any primary series vaccinations (oral polio vaccine, pneumococcal conjugate vaccine and the pentavalent vaccine) by 10 mo of age.

^II^Bolded risk factors are those considered risk factors of interest based on the univariate analysis and hypothesis.

^III^Sex and mother's age at birth were included in all crude analyses as variables a priori.

^IV^p-values obtained using Wald test.

^V^Adjusted for sex, mothers age at birth, distance from health centre, presence of parents, immigration and headship.

^VI^Adjusted for sex, mother's age at birth, ethnicity, presence of parents, immigration and headship.

^VII^Adjusted for sex, mother's age at birth, ethnicity, distance from health centre, presence of parents and headship.

^VIII^Adjusted for sex, mother's age at birth, ethnicity, immigration and distance from health centre.

^IX^Adjusted for sex, mother's age at birth, ethnicity, distance from health centre and immigration.

Cox proportional hazards models evaluated the association between vaccination status at age 10 mo and all-cause mortality between 10 and 24 mo of age as exploratory analyses. The analysis was repeated for vaccination status at age 15 mo and incidence of death between 15 and 24 mo of age. Potential confounders were included in both models. The proportional hazards assumption was assessed graphically and tested using Schoenfeld residuals.

### Missing data and sensitivity analysis

Unenumerated children without a 14-digit ID were considered non-residents of the BHDSS and excluded from analysis. Covariates with significant missing data (missingness ≥10%) were removed from the primary analysis. Sensitivity analyses, using binomial logistic regressions, checked for evidence whether data were missing at random (MAR). Any evidence (p<0.05) of differentially missing data was considered not missing at random (NMAR). Data were cleaned, validated and analysed using Stata (version 15.1, StataCorp, College Station, TX, USA).

## Results

**Figure 1.**
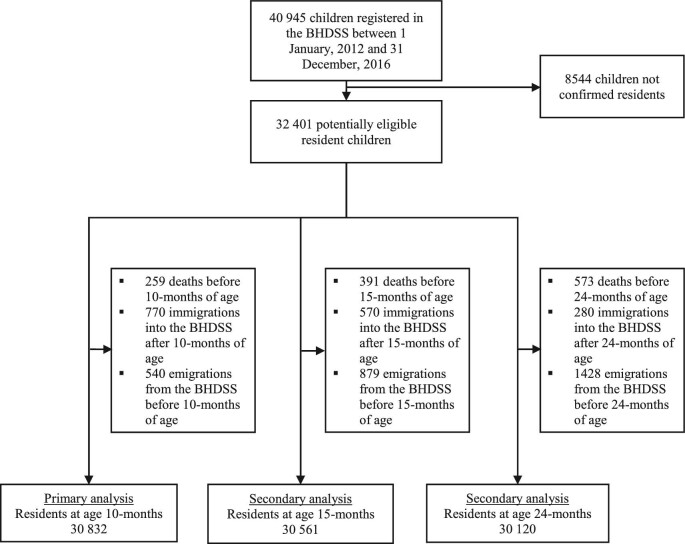
Flow diagram representing the total number of children registered, eligible and included for each of the three analyses at 10, 15 and 24 mo of age. The eligibility criteria required the participant to be a resident within the BHDSS at the respective age point of interest and born from 1 January 2012 to 31 December 2016. BHDSS, Basse Health and Demographic Surveillance system.

**Figure 2.**
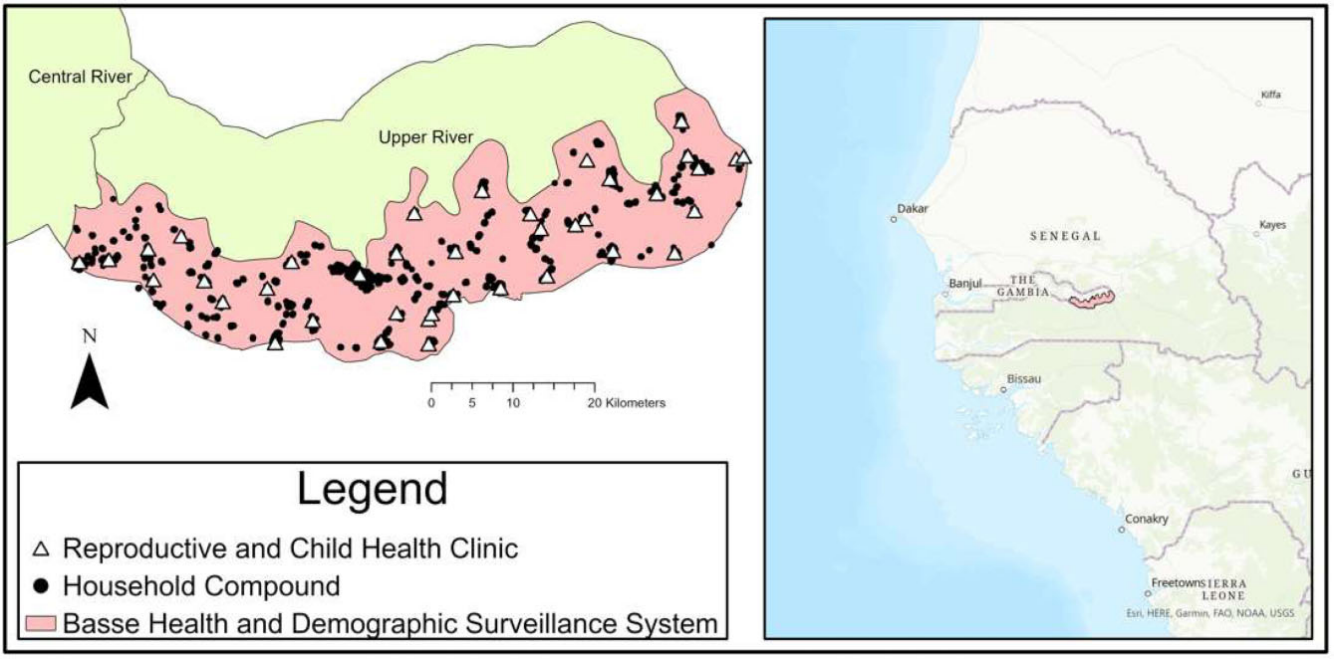
Map of household compounds for participants included in the study and the Reproductive and Child Health clinics in the Basse Health and Demographic Surveillance system, south of the Gambia River in the Upper River Region of eastern Gambia. Maps were created using ArcGIS® software by Esri. ArcGIS® and ArcMap™ are the intellectual property of Esri and are used herein under license. Copyright © Esri. All rights reserved. For more information about Esri® software, please visit www.esri.com.

There were 40 945 children registered in the BHDSS with birthdates from 1 January 2012 to 31 December 2016; 8544/40 945 (20.9%) were not confirmed residents and were excluded. Of the potentially eligible children, 30 832/32 401 (95.1%) were resident in the BHDSS at 10 mo of age. Figure 1 shows a flowchart of eligible children. Children were grouped into 22 153 families within 8314 households (median of four families per household and two children per family); 493 (1.6%) children were missing family data. Figure 2 represents the location of household compounds in the BHDSS.

At 10 mo of age, 1567/30 832 (5.1%) resident children were unvaccinated with the primary series. At 15 mo of age, 6886/30 561 (22.5%) of children were unvaccinated with the secondary series and 981/30 210 (3.3%) were unvaccinated at 24 mo of age with both primary and secondary series.

The distributions of risk factors at 10 mo of age are shown in Table 1. Most children (49.0%, 15 133/30 382) lived <0.5 km from an RCH clinic. Only 1170/30 382 (3.8%) children had experienced any type of migration before 10 mo of age. The majority (50.6%, 15 605/30 382) of children resided solely with their mother. The distributions of risk factors for the 15- and 24-mo vaccination status analysis are found in Supplementary Tables A2 and A3.

The results of the primary univariate analyses can be found in Table 1. At 10 mo of age, there was significant evidence that being unvaccinated with the primary series was associated with distance from an RCH clinic, presence of parents, household headship, migration status and ethnicity. The household's wealth quintile, sex, pregnancy type, birth order and mother's age at birth were not associated with vaccination status at 10 mo of age (Table 1). There were similar associative patterns observed at age 15- and 24-mo vaccination status in the secondary univariate analysis (Supplementary Tables A2 and A3). An important difference was the evidence (p<0.001) for an association between birth order and vaccination status at age 15 and 24 mo of age.

At 10 mo of age, after adjusting for confounders and clustering there was strong evidence (p<0.001) that Fula and Serahule children had higher odds of being unvaccinated compared with Mandinka children (Table 1, aOR 1.64, 95% CI 1.30 to 2.08 and 1.68, 1.35 to 2.09, respectively). There was weak evidence for increased odds of non-vaccination among children residing between ≥0.5–<1 km from an RCH clinic compared with <0.5 km (aOR 1.25, 95% CI 1.01 to 1.46) and no evidence of a difference for children residing other distances from RCH clinics (Table 1). After adjusting for confounders, children residing with a single mother had 38% higher odds of being unvaccinated than those residing with both parents (aOR 1.38, 95% CI 1.22 to 1.58); if they resided solely with the father the odds increased 2.94 times (aOR 2.94, 1.33 to 6.46). In the adjusted model, there remained strong evidence (p<0.001) for a deleterious effect on vaccination associated with external in-migration compared with no migration (aOR 2.60, 95% CI 1.39 to 4.87). Compared with non-migrants, there was no evidence (p=0.38) that children who internally in-migrated, and strong evidence (p=0.01) for those who migrated within the BHDSS, of an increase in odds of being unvaccinated (Table 1). In the adjusted model, there remained strong evidence (p<0.001) that a child without a parent as the head of the household had 29% higher odds of being unvaccinated compared with a child with either parent holding the position (aOR 1.29, 95% CI 1.09 to 1.52). The secondary multivariate analysis of unvaccinated status at age 15 and 24 mo showed similar findings (Supplementary Tables A2 and A3).

The Cox proportional hazards model showed no evidence of an association (p=0.34) between vaccination status at age 10 mo and mortality between 10 and 24 mo of age (adjusted HR [aHR] 1.25, 95% CI 0.79 to 2.00). There was strong evidence (p<0.001) of an association between 15-mo vaccination status and mortality between age 15 and 24 mo (aHR 1.81, 95% CI 1.32 to 2.48). Both 10- and 15-mo vaccination status survival models satisfied the proportional hazards assumption (Supplementary Figures A1 and A2).

In the primary dataset, 9259/30 832 (30.0%) of children were missing the household wealth quintile, and the variable was excluded from analysis. Wealth data were MAR across the outcome with no association between missingness and vaccination status (p=0.29). Education level was missing for 62% of mothers and 83% of fathers and was excluded from analysis. The data were NMAR and children with missing mother and father education data had increased odds of non-vaccination (cOR 1.18, 95% CI 1.06 to 1.31 and 1.41, 1.21 to 1.63, respectively).

## Discussion

We found that 1567/30 832 (5.1%) resident children at age 10 mo had missed all their primary series vaccines. There was strong evidence that children who (1) had at least one parent missing, (2) had experienced external in-migration, (3) whose parents were not the head of household or (4) were not Mandinka, had increased odds of being unvaccinated with their primary series compared with their counterparts.

We found that distance to an RCH clinic, a spatial issue, to have a minimal effect on non-vaccination. Distance to an immunisation clinic has been found to be associated with under-vaccination,^21,23,24^ and large distances impede immunisation uptake,^23^ which was negligibly reflected in our results. In settings where shorter distances are involved, such as rural Gambia, the hinderance from distances on vaccination may not be as prolific.

Literature also suggests increasing birth order is associated with delayed immunisation,^2^ whereas our primary analysis found no association with non-vaccination. Increasing birth order may make busier parents, a temporal issue, but not impede awareness or drive negligence towards receiving immunisation. In The Gambia factors related to spatial or temporal issues may associate more with delayed or missed immunisations; alternatively factors related to programme awareness, migration or levels of social care for vulnerable children may associate more with complete non-vaccination.

A Turkish study found an association between non-vaccination and in-migrant children, consistent with our findings in terms of external in-migration.^18^ This likely arises from a complex set of awareness or access issues: emigrating from areas without established immunisation programmes, lack of knowledge of the local immunisation programme or missing immunisation records.^18^ Our analysis found no evidence of a negative effect from internal in-migration or migration within the BHDSS. Immunisation coverage across The Gambia is quite stable and consistently recorded; Gambian children in-migrating may have had equal access to immunisations prior to departing their previous residential location.

Mandinka had a lower proportion of non-vaccinated children, which is consistent with previous literature.^11,25^ Ethnicity as a risk factor may derive from education and attitudinal issues and rural Serahule and Fula may be more doubtful of immunisation programmes.

Children living with one parent had higher odds of being unvaccinated, which is consistent with current literature.^26,27^ Single parenthood may combine temporal and awareness issues, where a caregiver is not available to take the child for immunisation, vaccines may be a lower priority for a busier parent,^16^ or with only one caregiver, they are less likely to be aware of immunisation programmes.

Children of parents who were not the household head had increased odds of being unvaccinated; this aligns with the current literature surrounding family structure and childcare in LMICs. A study in SSA found that mothers who were also the head of household were more likely to seek health facility care for their sick children compared with mothers who were not the head.^28^ Meanwhile, a Ghanian study found immediate children of the head had healthier diets relative to the other children in multi-family households.^29^ The importance of allocating resources to ensure a child receives their vaccinations may vary by child within households and be influenced by the head and their relationship to the child.

The lack of an association between the primary series status at 10 mo and mortality from 10 to 24 mo is understandable. The literature shows high immunisation coverage in the first year of life over the past 10 y in The Gambia and unvaccinated children benefit from herd immunity and the related reduction in vaccine-preventable deaths.^11^ The increased risk of mortality between 15 and 24 mo of age in children who remained unvaccinated with their secondary series may also be consistent with the literature, which suggests measles vaccine may have non-specific beneficial effects.^30^ Researchers have found a reduction in all-cause mortality of 30%–86% for children who received the standard measles immunisation.^30^

The size of our study meant that our rare endpoints were observed with sufficient frequency, allowing consideration of multiple covariates. Our study had >95% power to detect a 20% increase in odds, giving a low probability of type 1 error. The systematic enumeration in the BHDSS reduced selection bias via sampling error. As this was a population-based study, estimates of effect are representative of the population. Demographic data collected every 4 mo reduces measurement error in frequently changing variables and describes temporality. Our real-time electronic recording of immunisations eliminates recall and information bias.

Our study is limited by the variables collected by the BHDSS. Unmeasured risk factors could result in uncontrolled confounding. Factors such as perception of vaccine safety, polygamy or pregnancy outside of marriage may independently explain some of our observed effects. Over a quarter of the children had no SES data; these data were MAR and not associated with risk factors or vaccination status but a type II error is probable and its exclusion remains a limitation. Parental education level was deemed a likely confounder,^18^ however the data were largely missing (>65%). This remains a limitation and parents with varying education levels may explain some of the observed effect on children being unvaccinated at 10 mo of age.

The high proportion of registered children not confirmed resident in the BHDSS is unlikely to be a source of selection bias. It may be caused by neighbouring Senegalese families who cross the border to benefit from the Gambian EPI and children remain unenumerated (non-residents) as they falsify information at RCH clinics in fear of being excluded by immunisation officials.

Children who are unvaccinated may be more likely to be sick or unhealthy, and children who died before 10 mo of age were excluded from the analysis. Our analysis does not relate to such children. Our definition of in-migration only applied after a child's birth. This may bias the effect toward the null if the negative effect on non-vaccination lingers on immigrant parents despite the child being a native resident. In terms of presence of parents, the unknown status of the absent parent(s) may have unmeasured effects on the odds of vaccination, biasing the estimated effect towards the null.

## Conclusion

Our findings suggest that the factors that lead to unvaccinated children in The Gambia are multifaceted. It is unlikely that the noted limitations would completely remove the observed effects that these risk factors have on being unvaccinated. The generalisability of some risk factors may be limited by the unique characteristics of The Gambia, such as ethnicity. However, sociological factors such as parental presence, authorities within a household and immigration remain generalisable to other LMIC countries. EPI officials seeking to optimise their vaccination programmes should consider our findings, targeting the aforementioned risk factors to reduce rates of non-vaccination. Future studies should consider vaccine hesitancy, to better understand this trend in The Gambia and SSA.

## Supplementary Material

trac051_Supplemental_File

## Data Availability

The data underlying this article cannot be shared publicly due to the privacy of the participants in the study as the data identify individuals, families and residential location. Data may be shared upon reasonable request to the corresponding author and The Gambia Government/MRCG at LSHTM Joint Ethics Committee.
