# Supplementary material for: Risk factors associated with non-vaccination in Gambian children: a population-based cohort study
Source: Trans R Soc Trop Med Hyg. 2022 Jun 13;116(11):1063–70. doi: 10.1093/trstmh/trac051 (PMC9623738; doi:10.1093/trstmh/trac051)
Supplement: trac051_Supplemental_File [file trac051_supplemental_file.zip › Figure_A1_Supplementary_data.docx]

**Figure A1. Survival analysis using Nelson-Aalen cumulative hazard estimates on the incidence of all-cause mortality between 10- and 24-months of age dependent on the primary series vaccination status at 10-months of age for children in The Gambia. Children were considered unvaccinated if they missed all their doses of the primary series.**
