# Supplementary material for: Risk factors associated with non-vaccination in Gambian children: a population-based cohort study
Source: Trans R Soc Trop Med Hyg. 2022 Jun 13;116(11):1063–70. doi: 10.1093/trstmh/trac051 (PMC9623738; doi:10.1093/trstmh/trac051)
Supplement: trac051_Supplemental_File [file trac051_supplemental_file.zip › supplementary_content_TRSTMH_rev.docx]

**Supplementary content**

**Table A1**

*Secondary univariate analysis*

The secondary univariate analysis at 15- and 24-months of age generally followed the same patterns of association as with vaccination status at age 10-months (table A2 and table A3). There was strong evidence of an association between unvaccinated children and birth order at 15- and 24-months of age (p-value <0.001 and p-value=0.02, respectively). Third born children had 2.39 and 1.80 higher odds at age 15- and 24-months compared to first born children (cOR 2.39, 95% CI 1.94–2.95 and 1.80, 1.15 - 2.80).

There was no evidence in the univariate analysis of an association between distance of a residence from the RCH and the vaccination status at both 15- and 24-months of age (p=0.108 and p=0.435, respectively). There was no evidence for an association between the pregnancy type and the vaccination status at 15- and 24-months of age (p=0.19 and p=0.68, respectively). There was strong evidence for an association between the immigration status of a child at both 15- and 24-months of age and their vaccination status (p<0.001 and p=0.01). At both 15- and 24-months of age, children who experienced external in-migration had 70% and 175% higher odd of being unvaccinated, respectively, compared to children who had not experienced any in-migration (cOR 1.70, 95% CI 1.16–2.48 and 2.75, 1.55–4.87, respectively).

At 15- and 24-months of age there was strong evidence of an association between the ethnicity of the child and their respective vaccination status (p-value <0.001 & p-value <0.001). Mandinka ethnicity had the lowest proportion of unvaccinated children at age 15-months (16.9 %) and age 24-months (2.1%). Compared to Mandinka children, at 15-months of age Fula children had the largest increase in odds of non-vaccination, (cOR 1.74, 95% CI 1.65–1.93), at 24-months of age Fula and Serahule children had similar increased odds of non-vaccination compared to Mandinka children (cOR 1.64, 95% CI 1.30–2.08 and 1.68, 1.35–2.09, respectively).

Children whose parent(s) was not the head of household had higher odds of being unvaccinated at 15- and 24-months of age (cOR 1.11, 95% CI 1.01–1.21 and 1.32, 1.08–1.61, respectively). There was no evidence of an association between the mother’s age at birth and the vaccination status of the child at both 15- and 24-month age points of interest (p=0.12 and p=0.57, respectively). There was a strong association between the presence of parents and a child’s vaccination status at 15- and 24- months of age (p<0.001 and p<0.001, respectively). The crude analysis showed the greatest effect when comparing parameters to both parents being present in children with neither parent present whose odds of non-vaccination tripled and doubled, respectively (cOR 2.98, 95% CI 2.45–3.62 & 1.99, 1.38–2.87). There was no evidence of an association between the wealth quintile of the household and the vaccination status of the child at 15- and 24-months of age (p=0.67 and p=0.57, respectively).

**Table** **A2**

**Table A3**

*Secondary multivariate analysis*

The results for the multivariate analysis, adjusting for potential confounders and clustering, for the secondary outcomes, vaccination status at 15- and 24-months of age are found in table A2 and table A3. There remained strong evidence (p<0.001) that Mandinka children had lower odds of being unvaccinated compared to Fula and Serahule children at 15- and 24-months of age. In the adjusted models, there was some evidence that at 15- and 24-months of age, children who lived ≥0.5 and <1 km from a RCH had 12% and 25% higher odds of having missed their secondary series of vaccination compared to the children who lived <0.5 km (aOR 1.12, 95% CI 1.00–1.26 and 1.25, 1.01–1.55, respectively). There was no evidence for a difference in odds of being unvaccinated for children residing in any further distance parameters when compared to the nearest distance at both age points of interest (table A2 and table A2). There was strong evidence (p<0.001) at both age points of interest for higher odds of being unvaccinated if there was only a single mother or neither parent present compared to children whose parents were both present. At 15- and 24-months of age the greatest increase in odds of being unvaccinated when compared to a child whose parents were present was if neither parent were present, (aOR 2.98, 95% CI 2.45–3.62 and 1.99, 1.38–2.87, respectively). There was evidence for higher odds of being unvaccinated if a child experienced external migration compared to children who had not experienced any immigration, at age 15- and 24-months (aOR 1.75, 95% CI 1.12–2.59 and 2.46, 1.30–4.68, respectively). There were strong signs of negative confounding between model 1 and 2 for external migration at age 24-months as the point estimate increased by nearly 20% after adjusting for confounders (table A3). There was no evidence of a difference in odds of non-vaccination for children at age 15-months who had internally migrated compared to children who had not immigrated (aOR 1.19, 95% CI 0.93–1.51) as the CI’s included unity, however at 24-months of age there was evidence of a difference in odds between these parameters (aOR 1.53, 95% CI 1.01–2.29). After controlling for confounders there was some evidence of increased odds for being unvaccinated at age 24-months in children whose parent were not the head of house compared to children whose parents were (aOR 1.31, 95% 1.06–1.62).

*Death analysis*

The death analysis, using Cox proportional hazards models satisfied the proportional hazards assumption through graphical assessments (figure A1 & figure A2) and Schoenfeld residuals (p=0.72 and p=0.75, respectively).

**Figure A****1**

**Figure A2**
