# Supplementary material for: Risk factors associated with non-vaccination in Gambian children: a population-based cohort study
Source: Trans R Soc Trop Med Hyg. 2022 Jun 13;116(11):1063–70. doi: 10.1093/trstmh/trac051 (PMC9623738; doi:10.1093/trstmh/trac051)
Supplement: trac051_Supplemental_File [file trac051_supplemental_file.zip › Table_A1_Supplementary_data.docx]

**Table A1****. The Gambian vaccination schedule (2020).** ^1^

| Series | Age | Vaccine |
| --- | --- | --- |
| Birth | Birth | BCG, OPV1, HepB1 |
| Primary | 6–8 weeks | **DTP1**, **Hib1**, **HepB2**, **PCV1**, **OPV2**, Rota1 |
|  | 10–12 weeks | **DTP2**, **Hib2**, **HepB3**, **PCV2**, **OPV3**, Rota2 |
|  | 14–16 weeks | **DTP3**, **Hib3**, **HepB4**, **PCV3**, **OPV4**, IPV |
| Secondary | 9 months | **MR**, **Yellow Fever**, OPV5 |
|  | 12 months | MenA |
|  | 18 months | DTP4, MR2, |

^*^Bolded vaccines represent those measured in the current study. BCG: bacillus Calmette-Guerin vaccine, OPV: oral polio vaccine, HepB: Hepatitis b vaccine, DTP: diphtheria-tetanus-pertussis, Hib: conjugate *Haemophilus influenzae* type b vaccine, PCV: pneumococcal conjugate vaccine, Rota: rotavirus vaccine, MR: combination measles and rubella vaccine, MenA: group A meningococcal conjugate vaccine
