# Supplementary material for: Risk factors associated with non-vaccination in Gambian children: a population-based cohort study
Source: Trans R Soc Trop Med Hyg. 2022 Jun 13;116(11):1063–70. doi: 10.1093/trstmh/trac051 (PMC9623738; doi:10.1093/trstmh/trac051)
Supplement: trac051_Supplemental_File [file trac051_supplemental_file.zip › Table_A2_Supplementary_data.docx]

**Table A2. Secondary analysis of characteristics of children within the BHDSS and the crude and adjusted odds of being unvaccinated^I^ with the secondary vaccination series at 15-months of age. Selected risk factors of interest in bold.**

| Descriptive variable ^II^ | Total  N=30,561 | (Col %) | Unvaccinated  n=6,886 | (Row %) | Crude OR ^III^  (95% CI) | p- value ^IV^ | Adjusted OR (95% CI) | p-value ^IV^ |
| --- | --- | --- | --- | --- | --- | --- | --- | --- |
| Sex |  |  |  |  |  |  |  |  |
| Female | 14,943 | (48.9) | 3,365 | (22.5) | 1 | 0.12 | ND | ND |
| Male | 15,615 | (51.1) | 3,521 | (22.5) | 1.01 (0.95–1.07) |  |  |  |
| Missing | 3 | (0.0) | 0 | (0.0) |  |  |  |  |
| **Ethnicity** ^V^ |  |  |  |  |  |  |  |  |
| Mandinka | 6,365 | (20.8) | 1,075 | (16.9) | 1 | <0.001 | 1 | <0.001 |
| Fula | 9,717 | (31.8) | 2,424 | (24.9) | 1.74 (1.65–1.93) |  | 1.81 (1.62–2.02) |  |
| Serahule | 13,986 | (45.8) | 3,286 | (23.5) | 1.56 (1.41–1.72) |  | 1.56 (1.41–1.74) |  |
| Other | 490 | (1.6) | 101 | (20.6) | 1.29 (0.98–1.71) |  | 1.26 (0.93–1.70) |  |
| Missing | 3 | (0.0) | 0 | (0.0) |  |  |  |  |
| **Distance to RCH** ^VI^ |  |  |  |  |  |  |  |  |
| ≥0 & <0.5 km | 14,985 | (49.0) | 3,277 | (21.9) | 1 | 0.11 | 1 | <0.001 |
| ≥0.5 & <1 km | 4,565 | (14.9) | 1,072 | (23.5) | 1.14 (0.94–1.17) |  | 1.12 (1.00–1.26) |  |
| ≥1 & <2 km | 4,473 | (14.6) | 1,019 | (22.8) | 1.05 (0.94–1.17) |  | 1.01 (0.90–1.13) |  |
| ≥2 & <3 km | 3,012 | (9.9) | 694 | (23.0) | 1.07 (0.94–1.23) |  | 1.11 (0.97–1.27) |  |
| ≥3 & <4 km | 2,109 | (6.9) | 475 | (22.5) | 1.09 (0.94–1.26) |  | 1.12 (0.97–1.29) |  |
| ≥4 km | 274 | (0.9) | 55 | (20.1) | 0.82 (0.52–1.31) |  | 0.84 (0.54–1.32) |  |
| Missing | 1,143 | (3.7) | 294 | (25.7) |  |  |  |  |
| **Migration** ^VII^ |  |  |  |  |  |  |  |  |
| No in-migration | 27,736 | (90.8) | 6,145 | (22.2) | 1 | <0.001 | 1 | <0.001 |
| Within the BHDSS | 767 | (2.5) | 219 | (28.6) | 1.52 (1.25–1.84) |  | 1.39 (1.13–1.72) |  |
| Internal in-migration | 635 | (2.1) | 159 | (25.0) | 1.19 (0.94–1.51) |  | 1.19 (0.93–1.51) |  |
| External in-migration | 231 | (0.8) | 78 | (33.8) | 1.70 (1.16–2.48) |  | 1.75 (1.12–2.59) |  |
| Missing | 1,192 | (3.9) | 285 | (23.9) |  |  |  |  |
| Birth order |  |  |  |  |  |  |  |  |
| 1st | 21,996 | (72.0) | 4,626 | (21.0) | 1 | <0.001 | ND | ND |
| 2nd | 7,420 | (24.3) | 1,822 | (24.6) | 1.30 (1.21–1.40) |  |  |  |
| 3rd | 598 | (2.0) | 208 | (34.8) | 2.39 (1.94–2.95) |  |  |  |
| 4th or higher | 30 | (0.1) | 9 | (30.0) | 1.70 (0.65–4.42) |  |  |  |
| Missing | 517 | (1.7) | 221 | (42.7) |  |  |  |  |
| Pregnancy type |  |  |  |  |  |  |  |  |
| Singleton | 29,035 | (95.0) | 6,439 | (22.2) | 1 | 0.19 | ND | ND |
| ND Twins | 1,009 | (3.3) | 226 | (22.4) | 0.99 (0.79–1.24) |  |  |  |
| Missing | 517 | (1.7) | 221 | (42.7) |  |  |  |  |
| **Head of house** ^VIII^ |  |  |  |  |  |  |  |  |
| Was a parent | 6,044 | (19.8) | 1,279 | (21.2) | 1 | 0.04 | 1 | 0.08 |
| Was not a parent | 24,181 | (79.1) | 5,515 | (22.8) | 1.11 (1.01–1.21) |  | 1.09 (0.99–1.19) |  |
| Missing | 336 | (1.1) | 92 | (27.4) |  |  |  |  |
| Mothers age at birth |  |  |  |  |  |  |  |  |
| <15 | 176 | (0.6) | 36 | (20.5) | 1 | 0.12 | ND | ND |
| ≥15 & <20 | 3,720 | (12.2) | 789 | (21.2) | 1.11 (0.73–1.70) |  |  |  |
| ≥20 & <30 | 16,066 | (52.6) | 3,658 | (22.8) | 1.23 (0.81–1.85) |  |  |  |
| ≥30 & <40 | 8,740 | (28.6) | 1,892 | (21.6) | 1.12 (0.72–1.69) |  |  |  |
| ≥40 | 1,325 | (4.3) | 286 | (21.6) | 1.10 (0.71–1.70) |  |  |  |
| Missing | 534 | (1.7) | 225 | (42.1) |  |  |  |  |
| **Presence of parents ^XI^** |  |  |  |  |  |  |  |  |
| Both present | 13,672 | (44.7) | 2,693 | (19.7) | 1 | <0.001 | 1 | <0.001 |
| Father absent | 15,471 | (50.6) | 3,644 | (23.6) | 1.33 (1.24–1.43) |  | 1.35 (1.25–1.46) |  |
| Mom absent | 178 | (0.6) | 63 | (35.4) | 2.23 (1.42–3.49) |  | 2.14 (1.34–3.44) |  |
| Neither present | 1,240 | (4.1) | 486 | (39.2) | 2.98 (2.45–3.62) |  | 2.98 (2.43–3.66) |  |
| BHDSS: Basse Health and Demographic Surveillance system, OR: odds-ratio, RCH: reproductive and child health center.  ^I^ Children were defined as unvaccinated if they had not received any secondary series vaccinations (Measles and Yellow Fever) by 15 months of age.  ^II^ Bolded risk factors are those considered risk factors of interest based on the univariate analysis and hypothesis  ^III^ Sex and mothers age at birth and were included in all crude analyses as variables *a priori*  ^IV^ P-values obtained using Wald test  ^V^ Adjusted for sex, mothers age at birth, distance from health centre, presence of parents, immigration, headship, birth order and pregnancy type ^VI^ Adjusted for sex, mother's age at birth, ethnicity, presence of parents, immigration and headship ^VII^ Adjusted for sex, mother's age at birth, ethnicity, distance from health centre, presence of parents, headship, birth order and pregnancy type ^VIII^ Adjusted for sex, mother's age at birth, ethnicity and distance from health centre  ^IX^ Adjusted for sex, mother's age at birth, ethnicity, distance from health centre, immigration, birth order and pregnancy type | | | | | | | | |
