# Supplementary material for: Risk factors associated with non-vaccination in Gambian children: a population-based cohort study
Source: Trans R Soc Trop Med Hyg. 2022 Jun 13;116(11):1063–70. doi: 10.1093/trstmh/trac051 (PMC9623738; doi:10.1093/trstmh/trac051)
Supplement: trac051_Supplemental_File [file trac051_supplemental_file.zip › Table_A3_Supplementary_data.docx]

**Table A3. Secondary analysis of characteristics of children within the BHDSS and the crude and adjusted odds of being unvaccinated ^I^ with the secondary vaccination series at 24-months of age.** **Selected risk factors of interest in bold.**

| Descriptive variable ^II^ | Total  N=30,120 | (Col %) | Unvaccinated  (n=918) | (Row %) | Crude OR  (95% CI) ^III^ | p-value ^IV^ | Adjusted OR  (95% CI) ^IV^ | p-value ^IV^ |
| --- | --- | --- | --- | --- | --- | --- | --- | --- |
| Sex |  |  |  |  |  |  |  |  |
| Female | 14,704 | (48.8) | 446 | (3.0) | 1 | 0.57 | ND | ND |
| Male | 15,413 | (51.2) | 472 | (3.1) | 1.04 (0.90–1.21) |  |  |  |
| Missing | 3 | (0.0) | 0 | (0.0) |  |  |  |  |
| **Ethnicity** ^V^ |  |  |  |  |  |  |  |  |
| Mandinka | 6,262 | (20.8) | 130 | (2.1) | 1 | <0.001 | 1 | <0.001 |
| Fula | 9,536 | (31.7) | 307 | (3.2) | 1.64 (1.30–2.08) |  | 1.69 (1.32–2.16) |  |
| Serahule | 13,850 | (46.0) | 462 | (3.3) | 1.68 (1.35–2.09) |  | 1.67 (1.33–2.09) |  |
| Other | 469 | (1.6) | 19 | (4.1) | 2.33 (1.36–3.99) |  | 1.04 (0.89–1.20) |  |
| Missing | 3 | (0.0) | 0 | (0.0) |  |  |  |  |
| **Distance to RCH** ^VI^ |  |  |  |  |  |  |  |  |
| ≥0 & <0.5 km | 14,747 | (49.0) | 420 | (2.8) | 1 | 0.44 | 1 | <0.001 |
| ≥0.5 & <1 km | 4,526 | (15.0) | 155 | (3.4) | 1.25 (1.01–1.55) |  | 1.25 (1.01–1.56) |  |
| ≥1 & < 2 km | 4,421 | (14.7) | 139 | (3.1) | 1.11 (0.89–1.38) |  | 1.16 (0.92–1.48) |  |
| ≥2 & < 3 km | 2,973 | (9.9) | 96 | (3.2) | 1.16 (0.90–1.50) |  | 1.24 (0.96–1.62) |  |
| ≥3 & < 4 km | 2,069 | (6.9) | 56 | (2.7) | 0.96 (0.69–1.33) |  | 1.04 (0.75–1.43) |  |
| ≥4 km | 269 | (0.9) | 10 | (3.7) | 1.11 (0.52–2.34) |  | 1.06 (0.48–2.35) |  |
| Missing | 1,115 | (3.7) | 42 | (3.8) |  |  |  |  |
| **Migration** ^VII^ |  |  |  |  |  |  |  |  |
| No in-migration | 26,746 | (88.8) | 783 | (2.9) | 1 | 0.01 | 1 | <0.001 |
| Within the BHDSS | 1,033 | (3.4) | 41 | (4.0) | 1.44 (1.01–2.07) |  | 1.40 (0.95–2.06) |  |
| Internal in-migration | 849 | (2.8) | 36 | (4.2) | 1.59 (1.07–2.37) |  | 1.53 (1.01–2.29) |  |
| External in-migration | 300 | (1.0) | 19 | (6.3) | 2.75 (1.55–4.87) |  | 2.46 (1.30–4.68) |  |
| Missing | 1,192 | (4.0) | 39 | (3.3) |  |  |  |  |
| Birth order |  |  |  |  |  |  |  |  |
| 1st | 21,579 | (71.6) | 594 | (2.8) | 1 | 0.016 | ND | ND |
| 2nd | 7,333 | (24.3) | 246 | (3.4) | 1.27 (1.08–1.51) |  |  |  |
| 3rd | 597 | (2.0) | 27 | (4.5) | 1.80 (1.15–2.80) |  |  |  |
| 4th or higher | 28 | (0.1) | 2 | (7.1) | 2.71 (0.49–15.06) |  |  |  |
| Missing | 583 | (1.9) | 49 | (8.4) |  |  |  |  |
| Pregnancy type |  |  |  |  |  |  |  |  |
| Singleton | 28,538 | (94.7) | 837 | (2.9) | 1 | 0.68 | ND | ND |
| Twins | 999 | (3.3) | 32 | (3.2) | 1.09 (0.67 - 1.76) |  |  |  |
| Missing | 583 | (1.9) | 49 | (8.4) |  |  |  |  |
| **Head of house** ^VIII^ |  |  |  |  |  |  |  |  |
| Was a parent | 6,005 | (19.9) | 143 | (2.4) | 1 | 0.002 | 1 | <0.001 |
| Was not a parent | 23,782 | (79.0) | 760 | (3.2) | 1.32 (1.08–1.61) |  | 1.31 (1.06–1.62) |  |
| Missing | 333 | (1.1) | 15 | (4.5) |  |  |  |  |
| Mothers age at birth |  |  |  |  |  |  |  |  |
| <15 | 165 | (0.5) | 5 | (3.0) | 1 | 0.57 | ND | ND |
| ≥15 & <19 | 3,625 | (12.0) | 108 | (3.0) | 1.00 (0.39–2.57) |  |  |  |
| ≥20 & <29 | 15,777 | (52.4) | 487 | (3.1) | 1.05 (0.41–2.64) |  |  |  |
| ≥30 & <39 | 8,643 | (28.7) | 230 | (2.7) | 0.88 (0.35–2.25) |  |  |  |
| ≥40 | 1,311 | (4.4) | 39 | (3.0) | 1.00 (0.37–2.67) |  |  |  |
| Missing | 599 | (2.0) | 49 | (8.2) |  |  |  |  |
| **Presence of parents** ^IX^ |  |  |  |  |  |  |  |  |
| Both present | 13,157 | (43.7) | 311 | (2.4) | 1 | <0.001 | 1 | <0.001 |
| Father absent | 15,249 | (50.6) | 511 | (3.4) | 1.46 (1.25–1.72) |  | 1.44 (1.22–1.71) |  |
| Mom absent | 221 | (0.7) | 8 | (3.6) | 1.39 (0.53–3.64) |  | 1.49 (0.55–4.03) |  |
| Neither present | 1,493 | (5.0) | 88 | (5.9) | 1.99 (1.38–2.87) |  | 2.01 (1.38–2.92) |  |
| BHDSS: Basse Health and Demographic Surveillance system, OR: odds-ratio, RCH: reproductive and child health center.  ^I^  Unvaccinated defined as not received primary (oral polio vaccine, pneumococcal conjugate vaccine and the pentavalent vaccine) and secondary series vaccinations (Measles and Yellow Fever) by 24 months of age  ^II^ Bolded risk factors are those considered risk factors of interest based on the univariate analysis and hypothesis  ^III^ Sex and mothers age at birth and were included in all crude analyses as variables *a priori*  ^IV^ P-values obtained using Wald test  ^V^ Adjusted for sex, mothers age at birth, distance from health centre, presence of parents, immigration, headship and birth order ^VI^ Adjusted for sex, mother's age at birth, ethnicity, presence of parents, immigration and headship ^VII^ Adjusted for sex, mother's age at birth, ethnicity, distance from health centre, presence of parents, headship, birth order and pregnancy type ^VIII^ Adjusted for sex, mother's age at birth, ethnicity, distance from health centre, immigration and birth order  ^IX^ Adjusted for sex, mother's age at birth, ethnicity, distance from health centre and immigration | | | | | | | | |
